# Bioactive Glass Microscaffolds Fabricated by Two‐Photon Lithography

**DOI:** 10.1002/adma.202504475

**Published:** 2025-04-24

**Authors:** Leonhard Hambitzer, Jan Mathis Hornbostel, Louise Roolfs, Richard Prediger, Sebastian Kluck, Kai Zheng, Cornelia Lee‐Thedieck, Frederik Kotz‐Helmer

**Affiliations:** ^1^ Laboratory of Process Technology NeptunLab Department of Microsystems Engineering (IMTEK) University of Freiburg Georges‐Köhler‐Allee 103 79110 Freiburg Germany; ^2^ Institute of Cell Biology and Biophysics Department of Cell Biology University of Hannover Herrenhäuser Straße 2 30419 Hannover Germany; ^3^ Engineering Research Center of Stomatological Translational Medicine & Jiangsu Key Laboratory of Oral Diseases Nanjing Medical University Nanjing 210029 China; ^4^ Freiburg Materials Research Center (FMF) University of Freiburg Stefan‐Meier‐Straße 21 79104 Freiburg Germany; ^5^ Glassomer GmbH In den Kirchenmatten 54 79110 Freiburg Germany

**Keywords:** bioactive glass, microscaffold, nanoparticles, tissue engineering, two‐photon lithography

## Abstract

Porous scaffolds made of bioactive glass (BG) are of great interest for tissue engineering as they can bond to bone rapidly and promote new bone formation. Pores and channels between 100 and 500 µm provide space for cell intrusion and nutrient supply, facilitating bone ingrowth and vascularization. Furthermore, smaller pores and structural features of a few microns in size influence cell behavior, such as adhesion and osteogenic differentiation. Additive manufacturing (AM) is well suited to fabricate such geometries. However, microstructuring BG is demanding and common AM techniques are unable to achieve features below 100 µm. In this work, two‐photon lithography (TPL) is used for the first time to structure BG with single‐micron features. A composite containing BG nanoparticles is structured using TPL and thermally processed to receive glass scaffolds. The glass used in this study demonstrates in vitro bioactivity in simulated body fluid (SBF) and cytocompatibility toward human mesenchymal stromal cells (MSCs), making it a suitable material for tissue engineering. This process will open a toolbox for a variety of existing BG particles to be shaped with features as small as 6 µm and will broaden the understanding of the influence of scaffold design on cell behavior.

## Graphical abstract

Microstructuring of bioactive glasses offers great potential to influence cell behavior for bone tissue engineering. By utilizing two‐photon lithography of a nanocomposite and thermal post‐processing, single‐micron features and complex structures are achieved. The processed glass showed in vitro bioactivity and cytocompatibility toward human mesenchymal stem cells, making it suitable for culturing such cell types.

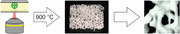

## 1 Introduction

BGs are used in biomedical applications ranging from bone tissue engineering to drug delivery.^[^ ^1^, ^2^ ^]^ They exhibit superior bonding to bone and promote new bone formation more efficiently than other inorganic biomaterials, such as calcium phosphates.^[^ ^3^, ^4^ ^]^ The osteogenic properties of BGs result from their dissolution products, mainly soluble silica and calcium ions.^[^ ^5^, ^6^ ^]^ The ionic microenvironment induces the precipitation of hydroxycarbonate apatite (HCA), the main inorganic component of bone.^[^ ^7^ ^]^ The newly formed HCA layer can adsorb proteins and attract bone marrow cells, leading to the formation of new bone.^[^ ^8^ ^]^ The ability to induce HCA precipitation is mainly dependent on surface chemistry, specifically the ability to release or bind calcium ions to the surface.^[^ ^9^ ^]^ BG is highly efficient in this regard due to its relatively high solubility compared to calcium phosphates and its ability to provide a favorable precipitation environment through the formation of a soluble silica layer during BG dissolution. This silica layer plays a crucial role in modulating the local ion concentration and pH, which further promotes the precipitation of mineralized phases. This mechanism is more efficient than that of materials that induce HCA precipitation by binding calcium on their surface and relying on the recruitment of ions from the surrounding environment, such as polydopamine.^[^ ^10^, ^11^ ^]^ Moreover, the amorphous structure of BGs offers significant advantages over crystalline calcium phosphates or polymeric materials, particularly when it comes to the incorporation of biologically active ions. The inherent flexibility of the amorphous network enables the facile doping of a wide range of ions, such as copper, zinc, and boron into the BG matrix.^[^ ^12^ ^]^ These ions play critical roles in various physiological processes, including the regulation of stem cell behavior and immune cell function.^[^ ^13^ ^]^ As a result, the inclusion of such active ions not only enhances the biological performance of BGs but also significantly broadens their biomedical applications, from promoting wound healing to advancing cancer therapy.^[^ ^6^ ^]^ Besides the chemical composition, the substrate geometry significantly influences cell behavior, such as cell adhesion, differentiation, and proliferation.^[^ ^14^, ^15^, ^16^, ^17^, ^18^ ^]^ While pores larger than 100 µm are considered essential for tissue ingrowth and metabolic transport,^[^ ^19^ ^]^ the presence of micropores smaller than 10 µm facilitates cell attachment and improves bone growth.^[^ ^14^, ^15^, ^20^, ^21^ ^]^ A highly microstructured and porous environment is found in cancellous bone, which is composed of a network of bone struts with widths ranging from 60–300 µm and a porosity of 50–90 %.^[^ ^22^ ^]^ It houses the microenvironment of the bone‐regenerating system, in which MSCs play a key role.^[^ ^23^ ^]^ Mimicking this microenvironment is a promising approach to enhance scaffold performance for bone tissue engineering or stem cell culturing.^[^ ^24^, ^25^ ^]^ However, fabricating such scaffolds is challenging and has primarily been achieved using biocompatible polymers, which do not possess the same level of bioactivity as BGs.^[^ ^26^ ^]^ Conventional methods such as casting, fiber spinning, sacrificial template, or foaming have been used for microstructuring BG, but lack geometric control or are restricted in the achievable feature sizes.^[^ ^27^, ^28^, ^29^, ^30^ ^]^ AM has emerged as a powerful tool for microfabrication, as it shapes parts with a high freedom of design. To structure BG, mostly indirect printing techniques like stereolithography,^[^ ^31^ ^]^ indirect selective laser sintering,^[^ ^32^ ^]^ binder jetting or robocasting have been used.^[^ ^33^, ^34^ ^]^ In these methods, BG particles are dispersed in a binder to form a composite, which is structured using AM and thermally processed to make pure glass. While these techniques allow great design freedom, the achievable resolution is limited. The smallest BG structures achieved by AM are struts with a width of ≈150 µm.^[^ ^35^ ^]^ This resolution limitation applies to the AM of bioactive ceramics as well. For example, the smallest beam lattice made of hydroxyapatite was printed with strut widths of 210 µm.^[^ ^36^ ^]^ Such structures have been predominantly fabricated using digital light projection, which typically uses pixel sizes >20 µm. The achievable feature size of the printed part is approximately four to ten times larger than the pixel size.^[^ ^37^, ^38^ ^]^ In addition, factors such as particle size, solid loading, and optical properties of the composite affect the resolution that can be achieved, often resulting features significantly larger compared to the nominal resolution of the printing system.^[^ ^39^ ^]^ However, this is an order of magnitude larger than many cells, such as osteoblasts or MSC, which diameter range between 12 and 50 µm.^[^ ^40^, ^41^ ^]^ It results in cell behavior more similar to 2D cultivation instead of what is observed in 3D in regards to cell contact to the material. To date, the limited resolution for structuring BG has hindered experimental insights into the influence of its microstructure on cell behavior. TPL is an AM technique capable of achieving structural resolution down to 100 nm.^[^ ^42^, ^43^ ^]^ It uses a photoresist that polymerizes upon absorption of at least two photons simultaneously. This process is nonlinear, therefore the reaction is confined to the focal volume of the laser, allowing structuring below the Abbe limit.^[^ ^44^ ^]^ Despite the high resolution and versatility, widespread applications have been limited by the low fabrication speed and the lack of suitable photoresists with high transparency at the processing wavelength. Recent technological advances have significantly increased the fabrication throughput, allowing the fabrication of centimeter‐sized parts.^[^ ^45^, ^46^ ^]^ Further improvements such as faster scanning systems and the usage of multiple beams are emerging.^[^ ^47^, ^48^ ^]^ TPL has been used to fabricate polymeric scaffolds to study the influence on cell behavior, including that of bone cells.^[^ ^16^, ^24^, ^49^, ^50^ ^]^ So far, TPL has been used to structure polymers, fused silica, or ceramics, which lack intrinsic bioactivity.^[^ ^51^, ^52^, ^53^, ^54^ ^]^ For application in bone tissue engineering, mainly polymeric or inorganic‐organic materials have been used.^[^ ^16^, ^55^, ^56^, ^57^ ^]^ The resins used in these studies rely on free radical polymerization, which can result in cytotoxicity by incomplete reaction of acrylates and initiator.^[^ ^58^ ^]^

In this work, we demonstrate for the first time shaping of BG using TPL, by utilizing a photocurable nanocomposite. The sol‐gel derived BG nanoparticles of this study serve as a representative model that can be extended to structure other established BG nanoparticles.^[^ ^12^ ^]^ Scaffolds were printed and subsequently converted into pure BG by a heat treatment. High‐resolution structures, such as a 2.5D pillar array with beam widths of 6 µm were achieved. We fabricated a variety of complex and integrated scaffolds in 3D, with features as small as 15 µm. Our work demonstrates that TPL is not restricted to small microscaffolds, but also allows structuring macroscopic scaffolds of up to 1 cm in length. This facilitates the fabrication of scaffolds with a practical size needed for cell culturing and tissue engineering. In vitro, the bioactivity of the received structures was demonstrated by the formation of HCA in SBF, which mimics the ion concentration of human blood plasma. It is an established method to indicate the bioactive behavior of BGs.^[^ ^59^, ^60^ ^]^ Furthermore, cytocompatibility was demonstrated by culturing immortalized human MSCs on BG. This novel method for fabricating BG microscaffolds allows advanced studies on the influence of BG microstructure on cell behavior.

## 2 Results and Discussion

**Figure 1:**
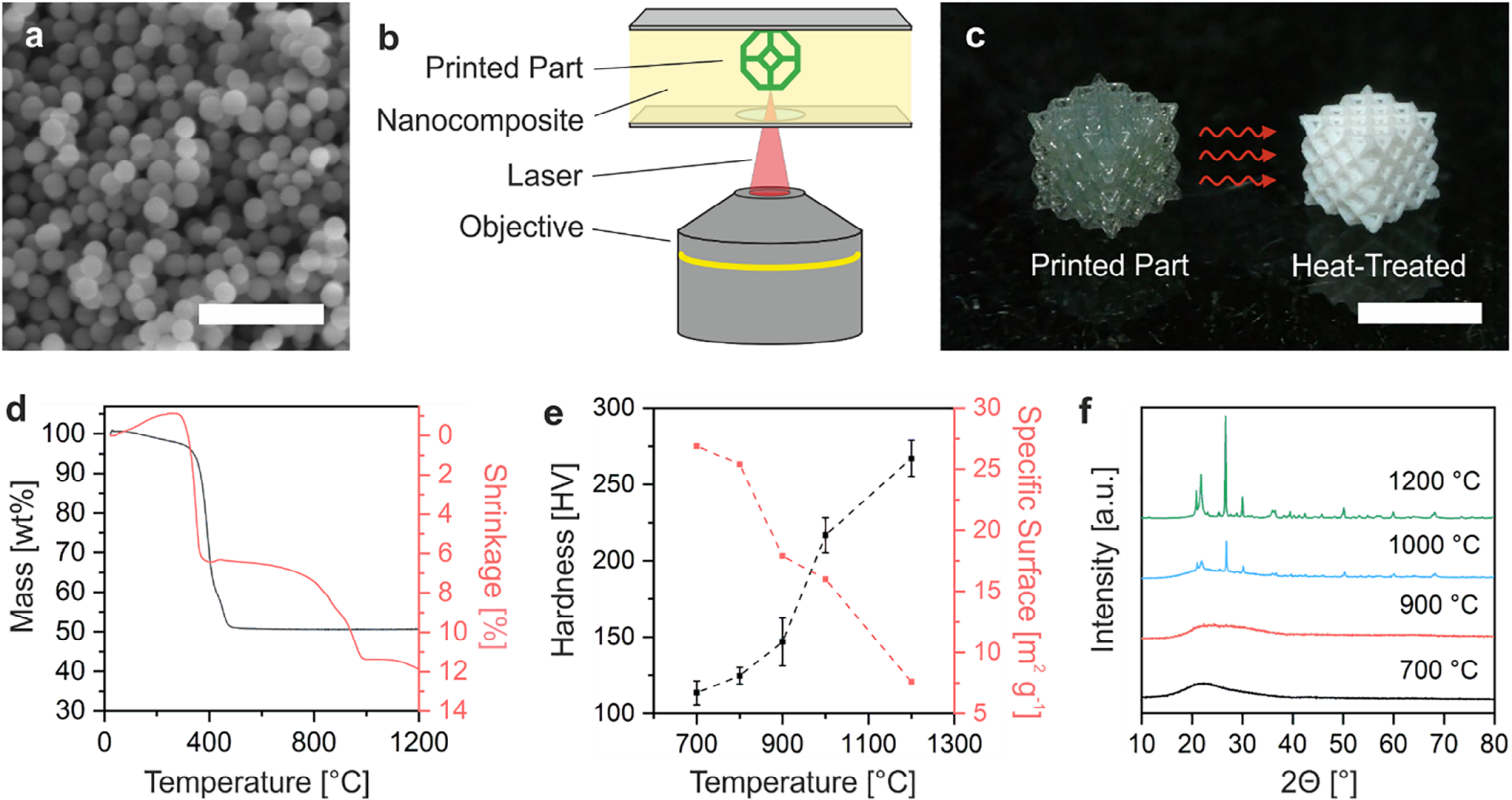
Microstructuring of BG using TPL. a) SEM image of the BG nanoparticles produced by a modified Stöber synthesis (scale bar: 500 nm). b) Schematic illustration of micro‐structuring BG with TPL inside a vat. c) After printing, the part underwent heat treatment at 900 °C to obtain pure BG structures. During the treatment, the organic binder decomposed and the part underwent shrinkage (scale bar: 1 mm). d) TGA showed that the decomposition of the binder was completed at 500 °C. Shrinkage during the heat treatment was characterized by dilatometry. e) An increase in Vickers hardness and a reduction of the surface area during heat treatment between 700 °C and 1200 °C was observed, indicating the solidification of the nanoparticles. f) XRD analysis revealed that crystallization occurs at temperatures above 900 °C.

For high‐resolution TPL, a photocurable composite with sufficiently small nanoparticles is required. To produce BG nanoparticles with a composition of 90 mol% SiO_2_ and 10 mol% CaO, we employed a modified Stöber synthesis using tetraethyl orthosilicate and calcium nitrate as precursors. This way, monodisperse nanoparticles with an average size of 110 nm and a specific surface area (BET) of 27 m^2^ g^−1^ (**Figure** **1**a) were obtained. Energy dispersive X‐ray analysis (EDX) confirmed the intended composition (Figure S1, Supporting Information). For the fabrication of the photocurable composite, a solid loading of 51 wt.% BG nanoparticles were dispersed in an acrylic binder. Citric acid was added as the dispersant to avoid gelation of the composite (Figure S2, Supporting Information). High optical transmission at the processing wavelength is key for achieving high‐resolution structuring. The nanoparticles need to be well dispersed as agglomeration leads to scattering. This was achieved by vigorous washing of the nanoparticles after synthesis with an ultrasonic lance, as excess reactant leads to agglomeration. The impact of the washing procedure was characterized by the optical transmission of a composite containing particles washed by stirring compared to one with particles washed using an ultrasonic lance (Figure S3, Supporting Information). The nanocomposite with particles washed using an ultrasonic lance demonstrated a high optical transmission of 90 % at 780 nm, which was the processing wavelength of the TPL system. This is a significant improvement compared to 57 % transmission of the nanocomposite prepared with particles washed by stirring. Structuring was done with the highly transparent composite using a NanoOne TPL system (UpNano GmbH) inside a vat (Figure 1b). After the printing process was completed, non‐polymerized resin was removed by immersion in a solvent. The printed parts were heat‐treated to receive pure BG structures (Figure 1c). The heat treatment should ensure full removal of the organic binder and improve the structure's mechanical performance while maintaining the amorphous nature of the BG. To determine the optimal heat treatment, the influence of temperature on these parameters was analyzed. Thermogravimetric analysis (TGA) was used to monitor decomposition during the heat treatment. Major decomposition occurred around 400 °C and was completed by 500 °C (Figure 1d). A mass of 51 wt.% remained, matching the initial weight of the BG and indicating complete removal of the binder. This process was accompanied by a reduction of the sample volume. A shrinkage of 6 % was measured with a dilatometer (Figure 1d). Further heating was applied to increase the mechanical performance by consolidation of the particles, which is favorable to allow facile handling and avoiding damage during cell cultivation. An additional 6 % shrinkage was observed for temperatures from 700 °C to 1200 °C. The specific surface area gradually decreased from 27 m^2^ g^−1^ at 700 °C to 8 m^2^ g^−1^ at 1200 °C, further indicating consolidation of the particles (Figure 1e). The influence of the processing temperature on the mechanical performance of the BG was measured by Vickers microindentation hardness, which characterizes the ability of a material to withstand deformation. The hardness increased from 114 HV for 700 °C to 267 HV for 1200 °C (Figure 1e). Therefore, a high temperature is beneficial for mechanical performance. However, the heat treatment should not lead to crystallization as it reduces the bioactivity of the BG.^[^ ^61^ ^]^ The crystallization during the heat treatment was studied by X‐ray diffraction (XRD) and the onset of crystallization was identified for temperatures over 900 °C (Figure 1f). The crystallization resulted in the formation of quartz, cristobalite, and wollastonite (Figure S4, Supporting Information). Therefore, 900 °C was chosen as the maximum processing temperature to achieve high mechanical performance, while maintaining the amorphous nature of BG. For this temperature, a linear shrinkage of 9 % and a surface area of 18 m^2^ g^−1^ were measured. The microporosity of the partially densified glass was analyzed using mercury porosimetry. A bulk density of 1.152 g cm^−1^ and a skeletal density of 2.290 g cm^−1^ were measured. Therefore, the glass maintained a porosity of 49.7 % at 900 °C. The hardness was 147 HV, which is higher than human bone, which typically ranges from 33–42 HV.^[^ ^62^, ^63^ ^]^ The value is significantly lower than hydroxyapatite with ≈600 HV and Bioglass 45S5 with ≈460 HV.^[^ ^64^ ^]^ This can be explained by the high porosity of the BG in this study, compared to the fully densified hydroxyapatite and Bioglas 45S5.

**Figure 2:**
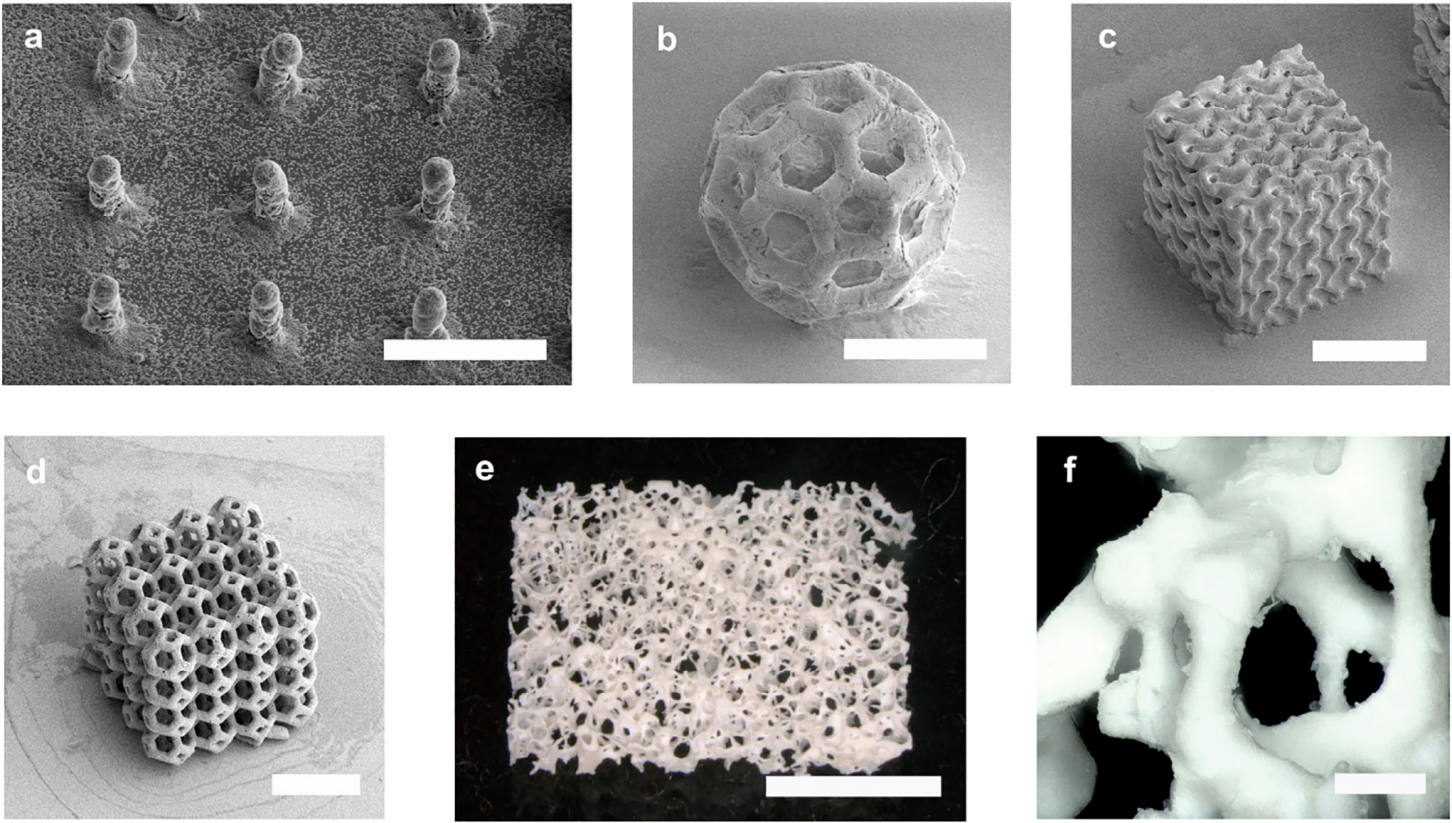
BG lattices fabricated using TPL and heat‐treated at 900 °C. a) 2.5D structures such as a pillar array with a beam width of 6 µm and a height of 10 µm (scale bar: 30 µm). b) A Buckyball with pores as small as 30 µm (scale bar: 100 µm). c) Gyroid lattice (scale bar: 150 µm), and d) truncated octahedron lattice (scale bar: 200 µm) show the variety of scaffolds that can be achieved. e) A model of human cancellous bone with 1 cm in length was fabricated (scale bar: 4 mm), which f) includes high‐resolution features (scale bar: 200 µm).

TPL of the nanocomposite in combination with the optimized heat treatment allowed high‐resolution structuring of amorphous BG. Single‐micron features were achieved for 2.5D structures, as can be seen from the pillar array fabricated with a height of 10 µm and width of 6 µm (**Figure** **2**a). Different 3D scaffolds show the versatility of the process. Complex and arching structures, such as a Buckyball with a beam width of 18 µm and pores as small as 30 µm, were successfully printed (Figure 2b). A gyroid lattice with wall thickness of 15 µm and porosity of 85 % was printed as a representative for triply periodic minimal surface structures (Figure 2c). This class of surfaces is of great interest for tissue engineering as they provide a high surface area and permeability.^[^ ^65^ ^]^ Furthermore, a truncated octahedron lattice with 83% porosity was fabricated (Figure 2d). Such truss‐type lattices have been widely investigated for their mechanical properties, which can be easily tailored.^[^ ^66^ ^]^ To demonstrate the capabilities of TPL to produce multiple millimeter‐sized structures with high resolution, a cancellous bone model derived from a µ‐CT scan of a human lumbar spine was printed (Figure 2e). The model had total dimension of 2.0 × 6.0 × 10.0 mm, struts as small as 63 µm, and a porosity of 88 % (Figure 2f). Fabricating scaffolds of such size facilitates handling for cell culturing and is of medical interest to treat critical bone defects. These are bone defects, that are too large to heal on their own and need the treatment with a bone graft.^[^ ^67^ ^]^ The process demonstrated a high shape fidelity within the margin of error between the nominal design and the printed part, exemplary examined for the octet lattices shown in Figure 1c (Figure S5 and Table S1, Supporting Information). Structuring with single‐micron resolution, such as the pillar array in Figure 2a showed distortions between the nominal and received shapes (Figure S6, Supporting Information). This indicates that achieving single micron comes with a trade of in‐shape fidelity.

**Figure 3:**
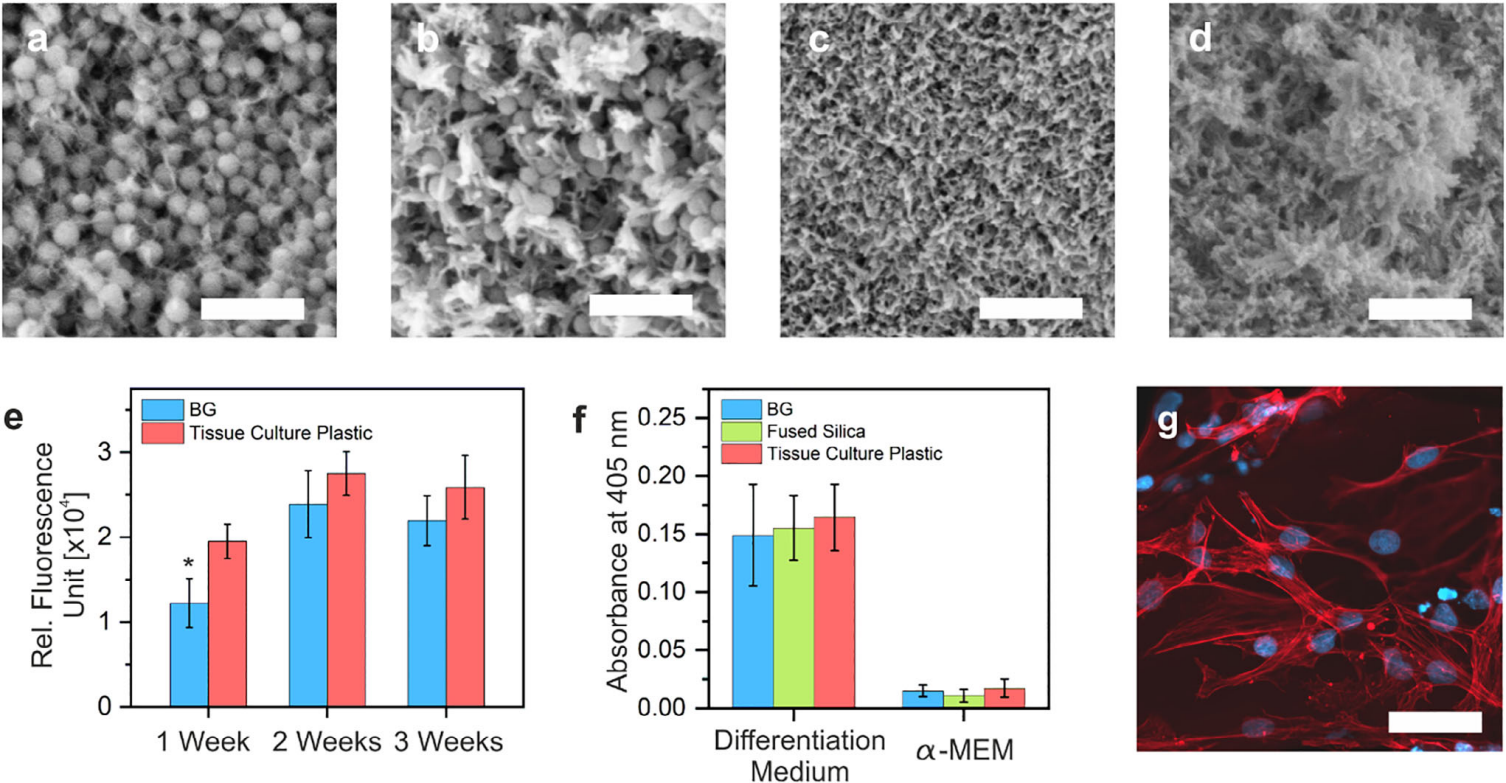
Biological characterization of BG after heat treatment at 900 °C. The in vitro mineralization of BG upon immersion in SBF was studied over the course of three weeks. Hydroxyapatite growth on the BG surface was investigated using SEM after a) 3 days, b) 1 week, c) 2 weeks, and d) 3 weeks (all scale bars, 500 nm). e) Cytocompatibility of BG was examined by a cell viability assay. Tissue culture plastic was used as the reference. Human mesenchymal stromal cells (iMSC#3) were seeded on BG bulk samples for three weeks and their cytocompatibility was confirmed. Data shows that the cell viability on BG is similar to tissue culture plastic. Data is presented as mean ± SEM, *n*  = 3, significant differences compared to the control are given with an asterisk * for *p* < 0.05 and were calculated with an unpaired Student's *t*‐test. f) Osteogenic differentiation of MSCs to osteoblasts was characterized using an ALP assay after three weeks. The BG allowed differentiation, comparable to fused silica and tissue culture plastic. Data is presented as mean ± SEM, *n*  = 3. g) Cell morphology was visualized by staining on cancellous bone scaffolds made of BG (scale bar: 60 µm).

The mechanical stability of the heat‐treated BG structures is of great importance for their application as in vitro cell scaffolds or potential in vivo implants. The stability was characterized by compression testing of microlattices. Octet lattices (3 × 3 × 3 unit cells) were printed and heat‐treated at 900 °C (Figure 1c). Taking into account the design porosity of 70 % and the bulk porosity of the glass, these lattices displayed a porosity of 81 %. The lattices were compressed until failure (Figure S7 and Table S2, Supporting Information). A compressive strength of 1.2 ± 0.3 MPa was measured, corresponding to a specific compressive strength of 2.8 ± 0.7 kN m kg^−1^. This is comparable to highly porous ceramics fabricated by TPL, such as alumina scaffolds with a specific strength of 0.3–24.6 kN m kg^−1^,^[^ ^68^, ^69^ ^]^ but significantly lower than the reported value of 121.6 ± 18.4 kN m kg^−1^ for fully densified silica glass.^[^ ^70^ ^]^ The specific strength of human cancellous bone varies greatly with sample preparation, geometric orientation, and location, but typically ranges from 2–56 kN m kg^−1^.^[^ ^71^, ^72^, ^73^, ^74^, ^75^, ^76^ ^]^ The BG scaffolds in this study fall in the lower range of this spectrum. While the stability of the heat‐treated BG structures was suitable for cell culture applications, their use as load‐bearing implants would require a different glass composition capable of achieving higher densification to ensure adequate stability. The ability to form HCA upon immersion in SBF was evaluated for the BG sintered at 900 °C over a period of three weeks. The surface of the samples was examined by SEM. Images representative of most of the surface are discussed (**Figure** **3**a–d). Initial crystal formation was observed after 3 days in the form of needle‐like crystals, which is typical of hydroxyapatite (Figure 3a).^[^ ^77^ ^]^ These crystals grew over the first week (Figure 3b) and completely covered the surface after two weeks (Figure 3c). Thereafter, the growth continued in an irregular manner (Figure 3d).^[^ ^78^ ^]^ After three weeks, the Ca/P ratio was 1.6, measured using energy dispersive X‐ray spectroscopy (EDX), which corresponds to stoichiometric hydroxyapatite (Figure S8, Supporting Information).^[^ ^79^ ^]^ In comparison, fused silica and acrylic polymer, both commercial and biocompatible TPL resins, did not show crystal growth over the first two weeks, with only minor crystal formation observed after three weeks (Figure S9, Supporting Information).

The suitability to use BG for cell culture was investigated by evaluating the viability, differentiation behavior, and cell morphology of MSCs. An immortalized human MSC line (iMSC#3) was selected, as it exhibits proliferation and differentiation behavior similar to native human stromal cells, including the ability to undergo osteogenic differentiation.^[^ ^80^ ^]^ A cell viability assay was performed and the metabolic activity of the MSCs was measured after 1, 2, and 3 weeks (Figure 3e). Tissue culture plastic served as the reference material. The viability of iMSC#3 was significantly (*p* < 0.0001) decreased after growth for 1 week on BG in comparison to the cells seeded on conventional tissue culture plastic before catching up with the viability of the control after 2 and 3 weeks. This temporary lower viability could be attributed to increased cellular adhesion and proliferation on tissue culture plastic surfaces. Similar to most cell lines, iMSC#3 was established and cultured on tissue culture plastic.^[^ ^80^ ^]^ This process yields a direct selection of cells with enhanced adhesion and proliferation on tissue culture plastic. Since roughness and topological cues of the cell environment were altered when seeding iMSC#3 cells on BG, they likely required time to adapt to the new conditions. Similar observations were made by Beijer et al., who showed that seeding MSCs which were precultured on tissue culture plastic onto topologically modified substrates initially leads to changes in cell morphology, reduced metabolic activity, and slower cell cycle progression.^[^ ^81^ ^]^ However, with time the cells were able to adapt to the new BG culture microenvironment, and thus, the tested BGs appeared cytocompatible and suitable for long‐time experiments such as differentiation assays.

The ability of MSCs to differentiate into the osteogenic lineage is a key characteristic of these cells with high relevance for tissue engineering. The differentiation behavior of iMSC#3 was investigated on structured samples mimicking cancellous bone. Fused silica with the same cancellous bone structure and tissue culture plastic served as the reference materials (Figure S10, Supporting Information). After three weeks of culturing, an alkaline phosphate (ALP) assay was performed, which is a well‐established method to evaluate the osteogenic differentiation capacity.^[^ ^82^ ^]^ The assay demonstrated that the BG supported osteogenic differentiation, with comparable results observed for the reference materials (Figure 3f). Similar observations were made when differentiating bone‐marrow derived MSCs on Bioglas 45S5 scaffolds.^[^ ^83^ ^]^ To show the tight interaction of the cells with the materials, the morphology of adherent cells was assessed via staining of the actin cytoskeleton and the nuclei after three weeks of culture (Figure 3g). A highly populated surface with elongated, spindle‐shaped cell morphology was observed on the struts of the printed structure mimicking cancellous bone, indicating excellent cell acceptance of the surface and tight cell‐material interactions. Despite the fact that the viability and the differentiation assay on BG did not show an advantage compared to the references, these results confirmed that BG has suitable properties for cell culture. Other metrics not included in this study, such as MSC gene expression or cell migration, have been reported to be positively affected by calcium doping in BGs and should be investigated in further studies.^[^ ^84^, ^85^ ^]^ The TPL structuring of BG allowed the fabrication of complex structures with high resolution and properties suitable for cell culture studies.

## 3 Conclusion

In this study, we demonstrate for the first time the high‐resolution structuring of BG by TPL. By using sol‐gel‐derived nanoparticles, this technology provides a way to structure a wide range of already established glass compositions. TPL allows the shaping of BG with features as small as 6 µm for 2.5D structures like pillar arrays, as well as 3D lattices with wall thicknesses of 15 µm. Further, we demonstrated the capability of TPL to produce high‐resolution scaffolds as large as 10 mm × 7.0 mm × 1.5 mm with features as small as 63 µm. The processed BG showed a superior ability to form HCA compared to fused silica and polymers, both commercial materials used in TPL. Cytocompatibility and support for osteogenic differentiation were confirmed for human MSCs. Although the BG did not outperform the reference materials in these cell assays, the results demonstrated its high bioactivity. This process allows the shaping of an important class of bioactive materials with single‐micron features. With the ability to create new, complex structures now possible, in‐depth insights into the interaction between cells with structured BG could be obtained. This is of great importance for tissue engineering and the advancement of cell culturing.

## 4 Experimental Section

### Materials

Tetraethyl orthosilicate, calcium nitrate tetrahydrate, citric acid, methanol, isopropyl alcohol, fetal bovine serum (FBS), phosphate‐buffered saline (PBS), penicillin, SIGMAFAST p‐Nitrophenyl phosphate (pNPP), puromycin and streptomycin were purchased from Sigma‐Aldrich (Germany). Ethanol and hydrochloric acid (37 % w:w) were purchased from Carl‐Roth (Germany). Toluene was purchased from Merck (Germany). 3‐methacyloxypropyl‐dimethylchlorosilane (MACS) and aqueous ammonia solution (28 % w:w) were purchased from abcr (Germany). Fused silica slides were purchased from Plano (Germany). Minimum Essential Medium (αMEM) was purchased from Pan‐Biotech (Germany) and cell wells were purchased from Greiner Bio‐One (Germany). Commercial resins, UpPhoto and UpQuartz, were purchased from UpNano (Austria). The acrylic binder containing a photoinitiator suitable for TPL was provided by Glassomer GmbH (Germany) Human Mesenchymal Stem Cell (hMSC) Osteogenic Differentiation Medium BulletKit was obtained from Lonza (Germany). DAPI was purchased from Thermo Fisher Scientific (Germany) and Phalloidin‐iFlour 488 from Abcam (United Kingdom).

### Nanoparticle Synthesis

The BG nanoparticles were synthesized using a modified Stöber synthesis.^[^ ^86^ ^]^ Briefly, 35 mL tetraethyl orthosilicate was dissolved in 140 mL ethanol. A solution of 50 mL aqueous ammonium hydroxide solution, 90 mL ethanol, and 290 mL deionized water was prepared and poured quickly in the previous solution under vigorous stirring. After 30 min, 4.2 g calcium nitrate tetrahydrate was added. Stirring was continued for a further 90 min respectively before the particles were collected by centrifugation at 8000 rpm for 5 min. The particles were washed twice by dispersing in deionized water and once in ethanol using an ultrasonic lance (UP200 St, Hielscher Ultrasonics, Germany) and collected by centrifugation at 8000 rpm after each washing step. The collected deposits were dried overnight at 65 °C before being calcinated at 650 °C for 1 h to remove the nitrate. A heating rate of 3 K min^−1^ was chosen. The nanoparticles were processed shortly after synthesis to produce the nanocomposite or otherwise stored in a desiccator over silica gel.

### Nanocomposite Preparation

The 1 wt.% citric acid was added as a dispersant to the acrylate‐based binder. 51 wt.% BG nanoparticles were dispersed stepwise into the binder using an ultrasonic lance. Cooling with an ice bath was performed to avoid overheating of the composite.

### Surface Functionalization

The fused silica slides were cleaned by immersion in acidic methanol (methanol:HCl, 1:1 v:v) for 30 min. Afterward, the slides were washed with isopropanol, deionized water, and then dried with nitrogen. Subsequently, the slides were immersed in a 100 mm solution of MACS in dry toluene for 60 min. Again, the slides were washed with isopropanol, followed by deionized water, and dried under nitrogen.

### Design of the Printing Files

The cancellous bone model was generated from a µCT scan of a human lumbar vertebra. The scan was provided as an open‐access file by the European Space Agency (ESA).^[^ ^87^ ^]^ The µCT data was processed into an STL file using 3D Slicer 5.0.3 (available under https://www.slicer.org).^[^ ^88^ ^]^ Other STLs of lattices were created with CAD software (Autodesk Inventor 2021, Autodesk Inc, USA).

### Two‐Photon Lithography

TPL was performed using a benchtop printing system (NanoOne, UpNano GmbH, Austria) in the vat mode. To ensure sufficient adhesion of the prints to the substrate, MACS‐functionalized fused silica slides were used. Depending on the anticipated feature size, either a 10× or 20× objective was chosen. For printing with the 10× air objective (NA 0.4, UPLXAPO10X, Olympus, Austria), a laser power of 140 mW at a scanning speed of 600 mm s^−1^ was used. Slicing in *x*‐ and *y*‐direction was done with a line distance (Δxy) of 0.3 µm and in *z*‐direction (Δ*z*) with 4.00 µm. With these settings, printing of the large cancellous bone model (10 mm × 7.0 mm × 1.5 mm, Figure 2e) took ≈8 h, the cancellous bone model for the ALP assay and immunostaining (5.0 mm × 3.5 mm × 1.5 mm, Figure S10, Supporting Information) took ≈1.5 h and the octet lattice for compression testing (500 µm × 500 µm × 500 µm, Figure S7, Supporting Information) took ≈10 min. For the 20× oil immersion objective (NA 0.7, UAPON20W340, Olympus, Austria), a laser power of 60 mW at a scanning speed of 300 mm s^−1^ was used. Δ*xy* was set to 0.25 µm and Δ*z* to 1.00 µm. Development was performed by immersion in ethanol for 15 min. UpPhoto and UpQuartz were printed using standard settings provided by the manufacturer. Thermal processing of the UpQuartz prints was done according to the recommended procedure of the manufacturer.

### Thermal Treatment

Removal of the organic binder and heat treatment of the scaffolds was done using an ashing furnace (AAF, Carbolite/Gero, Germany) in the air. A heating rate of 3 K min^−1^ and a dwelling phase of 1 h at the final processing temperature were applied. The sample was cooled to room temperature with 5 K min^−1^.

### Characterization

The morphology of the particles and the surface of the BG was characterized by scanning electron microscopy (SEM, Scios 2 DualBeam, Thermo‐Fischer Scientific, Germany). Dimensional measurements were performed with ImageJ.^[^ ^89^ ^]^ Compositional analysis of the nanoparticles was performed using an SEM equipped with an EDX spectrometer (Octane Elite EDS System, EDAX, Germany). Other microscopic imaging was performed with a digital light microscope (VHX6000, Keyence, Japan). The transparency of the photocurable nanocomposite in the range of 350–800 nm was measured using a UV‐vis spectrophotometer (Evolution 201, Thermo‐Fisher Scientific, Germany). A fused silica cuvette with a sample thickness of 1 mm was used. The viscosity was analyzed using a rheometer (MCR302, Anton Paar, Germany). The following characterization methods (BET, TGA, Dilatometry, Vickers hardness, and XRD) were performed on printed bulk samples with a diameter of 5 mm and a height of 2 mm. The specific surface area of the nanopowder and heat‐treated prints was measured using nitrogen adsorption according to Brunauer‐Emmett‐Teller (BET) method (Sorptomatic 1990, Thermo Electron Corporation, USA) and calculated using a three‐parameter fit. The thermal decomposition of the printed nanocomposite was characterized with TGA (STA 449F5, Netzsch, Germany). The measurement was performed from room temperature to 1200 °C in air using a heating rate of 3 K min^−1^. Changes in the sample dimension during the heat treatment were characterized with a dilatometer (DIL 402C, Netzsch, Germany) from room temperature to 1200 °C in the air with a heating rate of 1 K min^−1^ Vickers hardness was measured by microindentation (MHT‐10, Anton Paar, Germany). For this, the samples were embedded in an epoxide resin and polished to receive an even surface for the measurements. A load of 100 N was applied for 10 s. Each sample was measured 10 times and the results were averaged. XRD (D8 Discover, Bruker AXS GmbH, Germany) was measured in the 2θ‐range of 10–80° with Cu‐K_α_ radiation (0.154060 nm). The bulk and skeletal density were characterized by mercury porosimetry (Autopore V, Micromeritics Instrument Corporation, USA). The height profile of the micropillar array was measured using white light interferometry (NewView 9000, Zygo, USA). Microcompression testing was performed using a 5 N force sensor (XFTC300, TE connectivity, Germany) paired with a stepper motor featuring a resolution of 0.1 µm. A constant speed of 5 µm s^−1^ was used. Five samples were compressed until failure and the results averaged.

### In Vitro Mineralization

In vitro, the mineralization of BG was investigated by HCA formation in SBF, which was prepared according to Kokubo et al.^[^ ^90^ ^]^ The test follows the procedure recommended by the Technical Committee 4 (TC04) of the International Commission of Glass (ICG).^[^ ^59^ ^]^ 75 mg of the sample were immersed in 50 mL SBF. The solution was kept at 37 °C in a shaking incubator (Minitron, Infors‐HT, Germany) for up to three weeks. For analysis, the samples were washed with distilled water, dried at 60 °C for 24 h, and characterized by SEM and EDX.

### In Vitro Cell Testing

The metabolic activity of an immortalized human mesenchymal stromal cell line (iMSC#3) on heat‐treated BG was assessed using a colorimetric analysis employing the fluorescent redox dye resazurin (CellTiter‐Blue Cell Viability Assay, Promega GmbH, USA). The iMSC#3 cell line (RRID: CVCL_B5PE) was kindly provided by Prof. Dr. Ola Myklebost (University of Bergen, Department of Clinical Science, Norway). They were established by immortalization of human bone marrow MSCs by retroviral transduction of telomerase reverse transcriptase.^[^ ^80^ ^]^ The viability assay relies on the reduction of resazurin to resorufin, a process catalyzed by metabolically active cells, thus indicating cell viability. iMSC#3 cells were cultured in αMEM medium supplemented with 10 % FBS and 2 µg mL^−1^ puromycin which was replaced with 100 U mL^−1^ penicillin and 100 µg mL^−1^ streptomycin when using the cells for experiments. Disk‐shaped samples (2 × 5 mm) were prepared via casting and heat‐treated at 900 °C for 1 h. Before incubation, the samples were sterilized in 70 % ethanol for 10 minutes and washed three times with PBS. Subsequently, the samples were placed in a 48‐well plate and immersed in 500 µL of the cultivation media for 24 h. In order to compare between the manufactured BG samples and the commercially available tissue culture plastic, wells of the same plates were used as references. Both the samples and the reference were seeded with 1 × 10^4^ cells and cultured at 37 °C and 5 % CO_2_ in a humid atmosphere. The CellTiter‐Blue assay was performed after 1, 2, and 3 weeks. For the assay, 100 µL of the CellTiter‐Blue solution was added to the cell culture wells at a 1:5 ratio to the culture volume, followed by incubation for 3 h at 37 °C. Absorbance measurements were conducted in triplicates at 560 and 590 nm using a fluorescence plate reader (Infinite 200 PRO, Tecan, Switzerland). The viability tests were performed in triplicates (*n*  =  3). For the results of these experiments, the mean value of the data was calculated as well as the standard error. The statistical significance of the obtained data was determined using an unpaired Student's *t*‐test with GraphPad Prism software (GraphPad Software, USA). To differentiate iMSC#3 cells osteogenically, 1 × 10^4^ cells were seeded in αMEM medium supplemented with 10 % FBS on BG and fused silica scaffolds, which resembled human cancellous bone (Figure S9, Supporting Information). The scaffolds were prepared as described above. Tissue culture plastic was used as the reference in a wells of a 48‐well plate in duplicates (*n*  =  2). After 24 h, the medium was changed to a commercially available osteogenic differentiation medium (hMSC Osteogenic Differentiation Medium BulletKit). The cells were further cultured for 21 days following the manufacturer's instructions. Additionally, controls were prepared which were cultured in α‐MEM for the whole experiment. On day 21 of the differentiation, the supernatant was harvested and the alkaline phosphatase (ALP) activity was measured using pNPP as a substrate. For this, a pNPP‐stock was produced from SIGMAFAST tablets by solubilizing one pNPP‐ as well as one TRIS‐tablet in 2 mL PBS and 4 mL ultrapure H2O. 80 µL of the supernatants were transferred to the wells of a 96‐well plate in triplicates. Subsequently, 20 µL of the pNPP‐stock was added. The mixture was incubated for 1 h at 37 °C under light exclusion. The absorbance was then measured at 405 nm using the aforementioned plate reader. The experiment was repeated three times.

### Fluorescence Microscopy

In order to evaluate the morphology of the cells as well as the adherence on the scaffolds, the nuclei as well as the actin filaments of the cells were stained. For this, iMSC#3 cells were seeded onto BG and fused silica scaffolds, and differentiated as described above. The samples were then fixed for 15 min using 4 % PFA in PBS. After three subsequent washing steps with PBS for 5 min each, the cells were permeabilized by submerging the scaffolds in a 0.1 % TritonX 100 solution in H_2_O for 10 additional minutes. The cells were again washed three times and were then subjected to a filtered 1 % BSA in PBS solution overnight at 4 °C. After washing the cells three additional times, they were incubated in a solution containing DAPI (1 µg mL^−1^) and Phalloidin iFlour 488 (1:1000) in 0.1 % BSA in PBS for 1 h at RT. After the last washing step, the samples were imaged using a confocal laser scanning microscope (LSM 980, Carl Zeiss, Oberkochen, Germany).

## Conflict of Interest

The authors declare no conflict of interest.

## Supporting information



Supporting Information

## Data Availability

The data that support the findings of this study are available from the corresponding author upon reasonable request.
