# Supplementary material for: Bioactive Glass Microscaffolds Fabricated by Two‐Photon Lithography
Source: Adv Mater. 2025 Apr 24;37(29):2504475. doi: 10.1002/adma.202504475 (PMC12288830; doi:10.1002/adma.202504475)
Supplement: Supplementary file 1 — Supporting Information [file ADMA-37-2504475-s001.docx]

**Supporting Information**

Bioactive Glass Microscaffolds Fabricated by Two‑Photon Lithography

Leonhard Hambitzer, Jan Mathis Hornbostel, Louise Roolfs, Richard Prediger, Sebastian Kluck, Kai Zheng*, Cornelia Lee-Thedieck, Frederik Kotz Helmer*


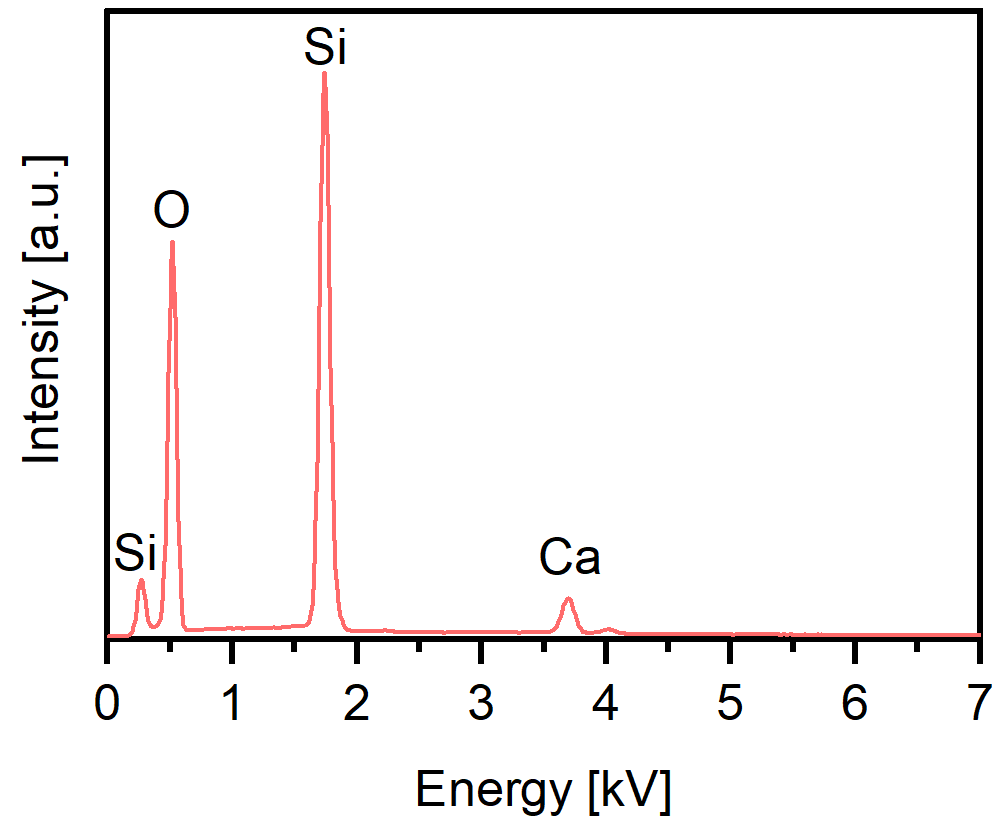


**Figure S1.** EDX analysis of sol-gel derived BG nanoparticles. The composition analysis of the BG after synthesis confirms the intended CaO content of 10 mol% and 90 mol% SiO_2_.


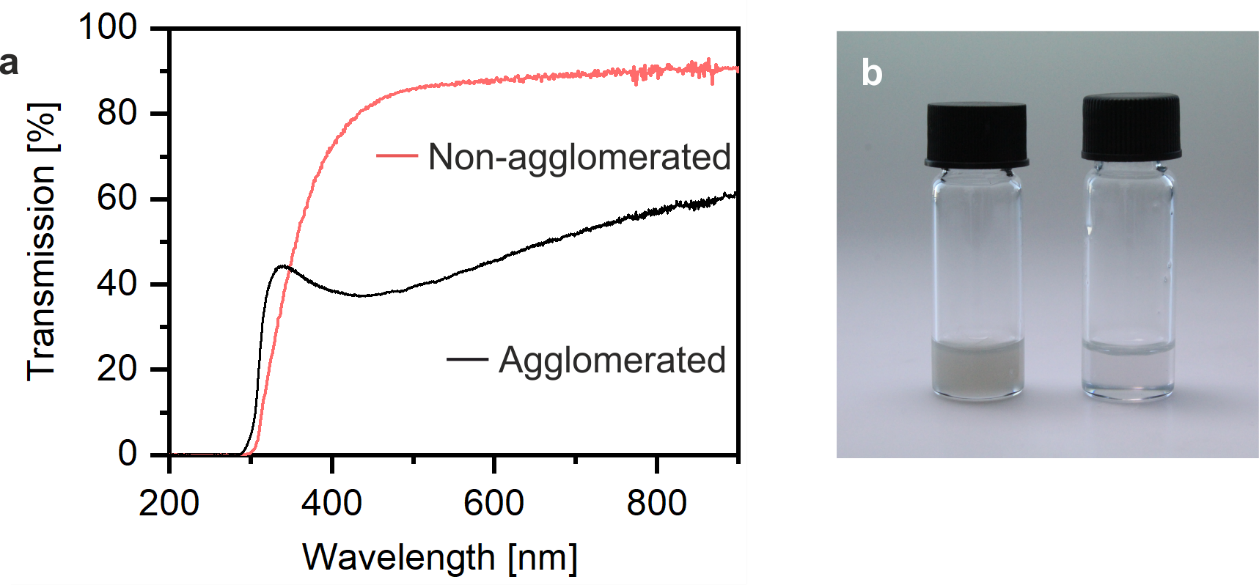


**Figure S2.** Effect of agglomeration on the transparency of BG nanocomposites. a) UV–Vis spectroscopy of nanocomposites prepared with agglomerated particles and non-agglomerated particles. Agglomeration of the nanoparticles, which was heavily influenced by the particle synthesis, strongly affected the transmission of the nanocomposites. Agglomerated particles were obtained by using stirring to wash the nanoparticles during synthesis. Non-agglomerated particles were obtained using an ultrasonic lance. b) Optical appearance of the nanocomposites with agglomerated particles (left) and non-agglomerated particles (right).


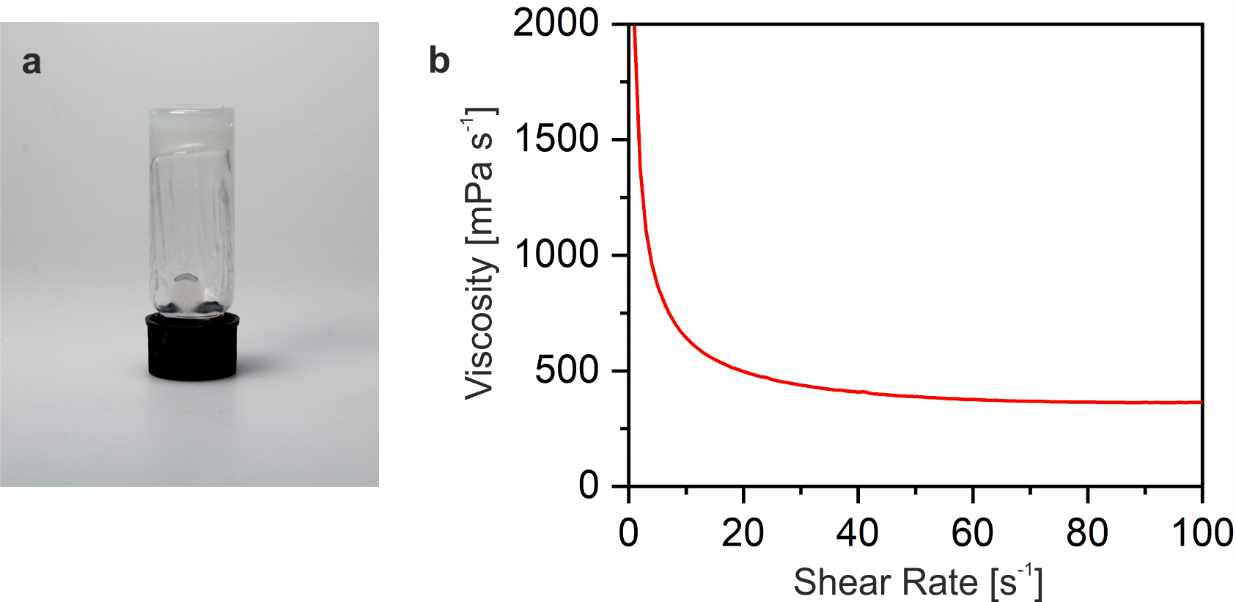


**Figure S3.** Rheology of the nanocomposite and the influence of the dispersant. a) Without citric acid as the dispersant, the resin turned into a gel. b) By adding citric acid, a free-flowing nanocomposite with shear-thinning behavior was obtained.


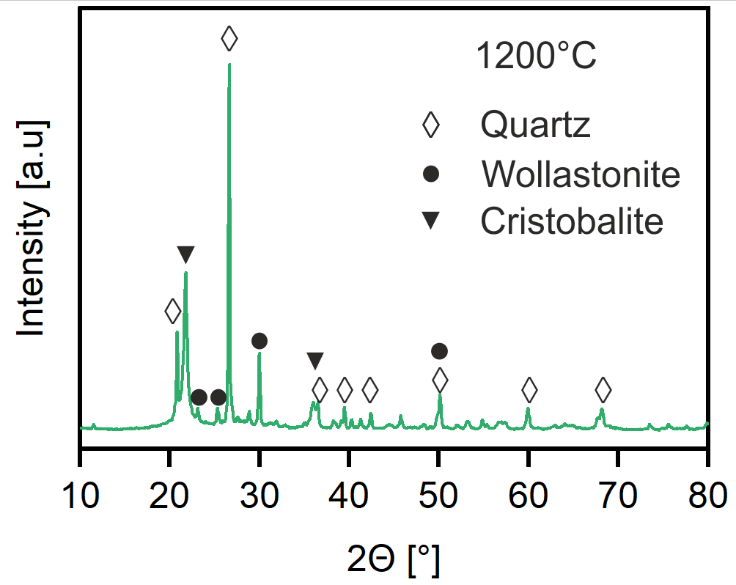


**Figure S4.** XRD analysis of BG heat-treated at to 1200 °C. The glass crystallizes to quartz (PDF 04-012-0490), wollastonite (PDF 04-016-5334) and cristobalite (PDF 04-008-7638).

**
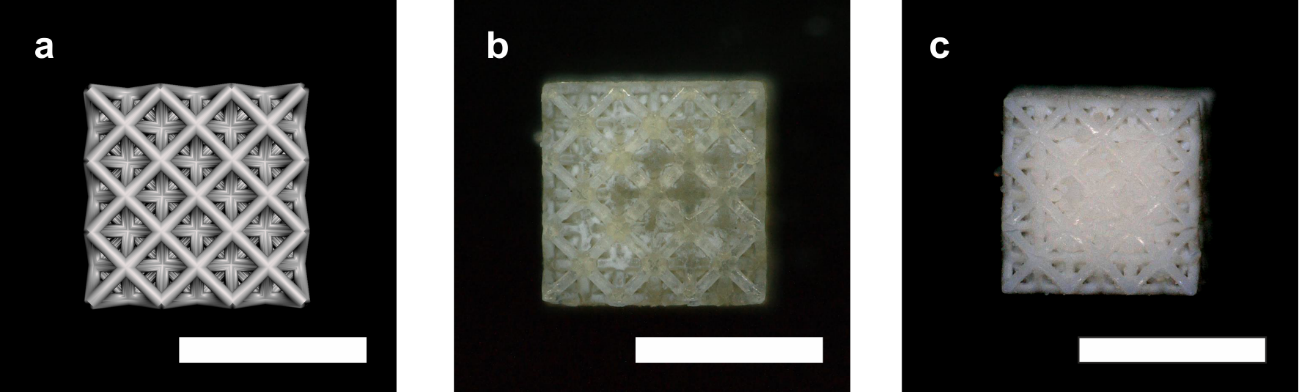
**

**Figure S5.** Exemplary examination of the process fidelity. a) STL design for the octet lattice shown in Figure 1c. b) The dimensions of the printed part demonstrated high shape fidelity compared to the STL design. c) Uniform shrinkage was observed after heat-treatment (all scale bars, 1 mm).

**Table S1**. Examination of the process accuracy of STL, printed part and heat-treated part. The overall scaffold width and diameters of the individual lattice beams were measured, each at 10 individual points.

|  | **Scaffold width [µm]** | **Lattice beam diameter [µm]** |
| --- | --- | --- |
| STL design | 1200 | 72 |
| Printed part | 1196 ± 5 | 71 ± 2 |
| Heat-treated | 1054 ± 7 | 64 ± 2 |


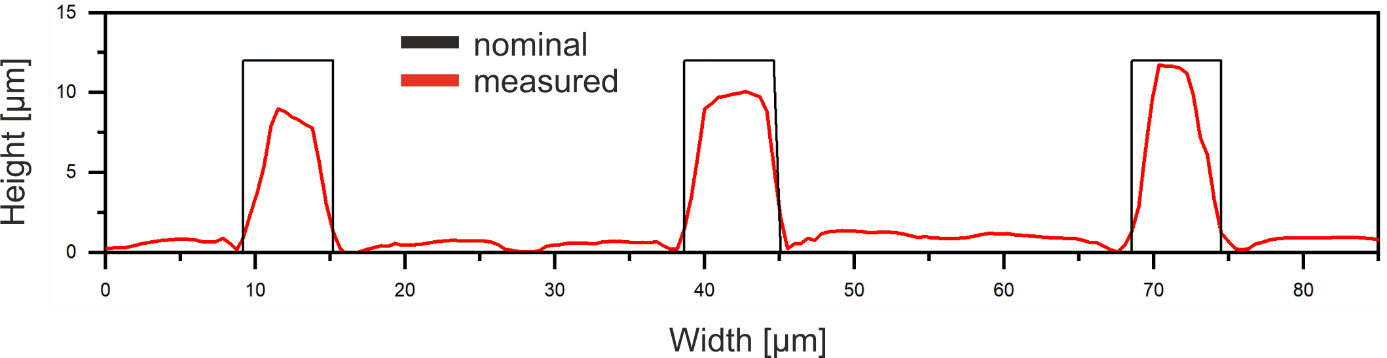


**Figure S6.** Exemplary height profile of the pillar array shown in Figure 2a measured using white light interferometry. The nominal and measured profiles are compared. The differences indicate that single micron structuring is the limit for this process, as structuring is possible, but with a tradeoff in shape fidelity.


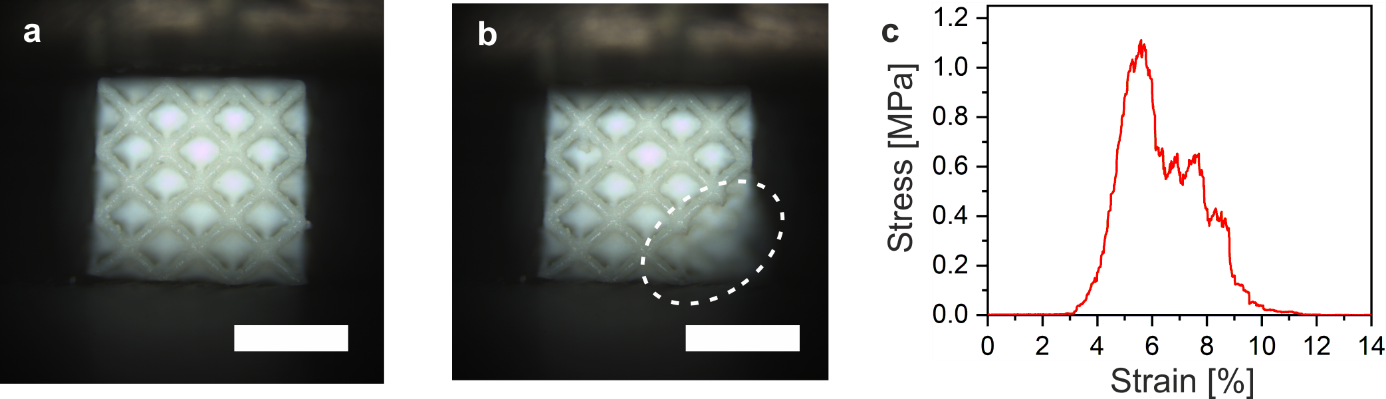


**Figure S7.** Exemplary images and stress-strain diagram of the compression test. a) Undamaged lattice during the beginning of the deformation. b) Failure in the corner of the lattice at the maximum stress. c) Typical stress-strain diagram showing a catastrophic failure.

**Table S2.** Parameters of TPL-printed octet lattices characterized by compression.

| Sample | Density [g m^‑3^] | Maximum Strength [MPa] |
| --- | --- | --- |
| 1 | 0.495 | 0.91 |
| 2 | 0.468 | 1.13 |
| 3 | 0.325 | 1.11 |
| 4 | 0.467 | 1.28 |
| 5 | 0.465 | 1.71 |


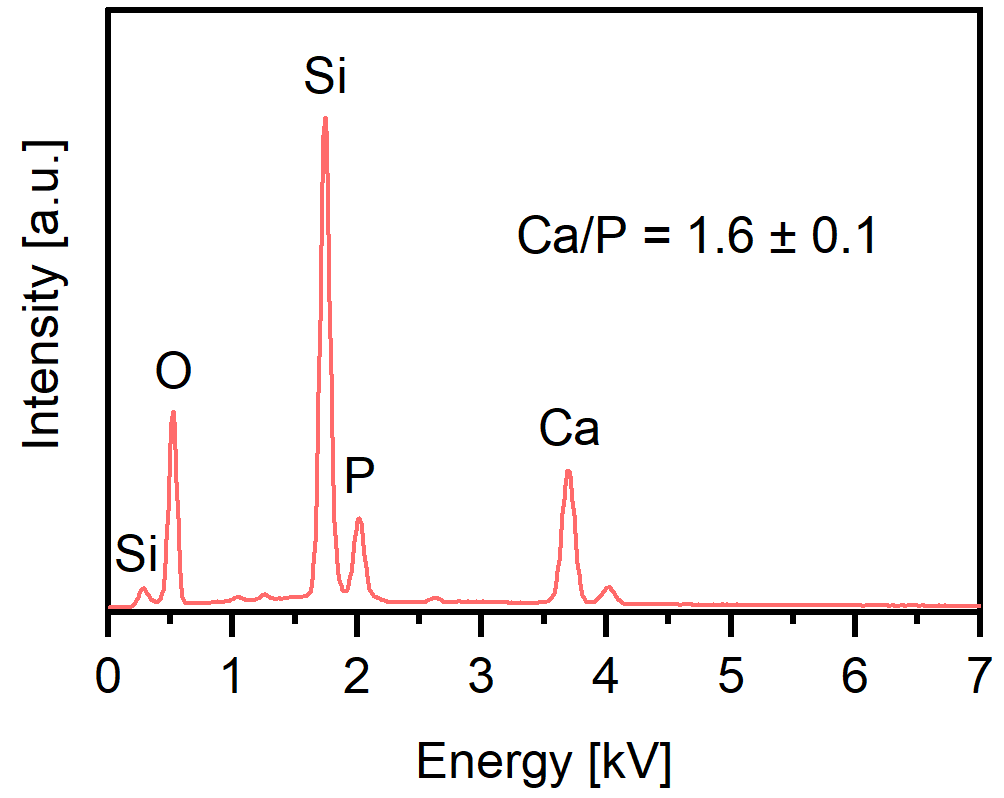


**Figure S8.** EDX analysis of the crystals formed on the surface of BG after immersion in SBF for 7 days. The BG was heat-treated at 900 °C, prior immersion. The calcium to phosphorus ratio of 1.6 ± 0.1 confirms the formation of HCA.


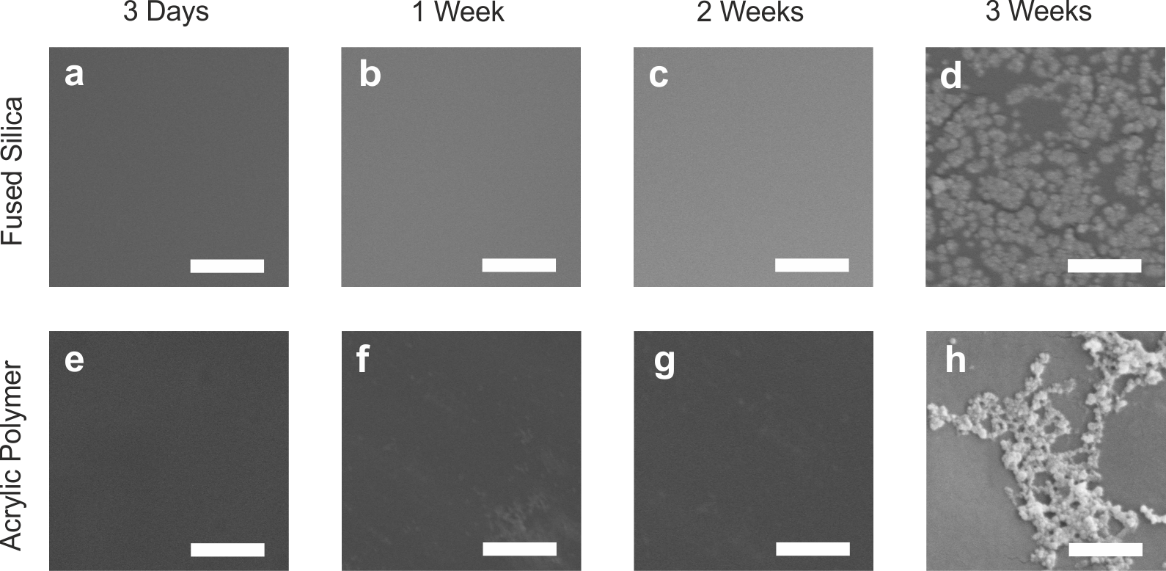


**Figure S9.** In vitro mineralization of fused silica and acrylic polymer, which were structured by TPL, upon immersion in SBF. Commercial resins were used and the final structures immersed in SBF for three weeks (all scale bars, 500 nm). a–d) Fused silica showed no crystallization within the first two weeks. e–h) Similar observation was made for the acrylic polymer structure as no crystallization was observed for two weeks. Only minor crystals were observed for both samples after three weeks.


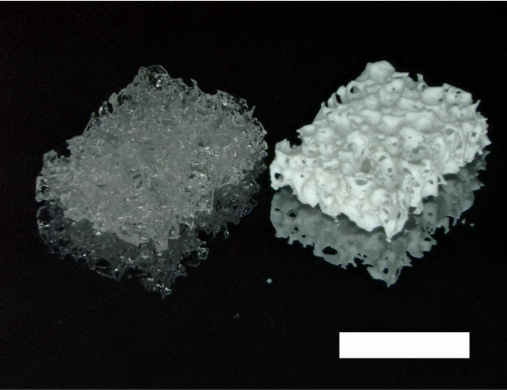


**Figure S10**. Cancellous bone scaffolds used for the ALP assay and cell staining. On the left side is a fused silica and right a heat-treated BG scaffold. Both scaffolds had a porosity of 74 %.
